# Building Vulnerability in a Changing Climate: Indoor Temperature Exposures and Health Outcomes in Older Adults Living in Public Housing during an Extreme Heat Event in Cambridge, MA

**DOI:** 10.3390/ijerph16132373

**Published:** 2019-07-04

**Authors:** Augusta A. Williams, John D. Spengler, Paul Catalano, Joseph G. Allen, Jose G. Cedeno-Laurent

**Affiliations:** 1 Department of Environmental Health, Harvard TH Chan School of Public Health, Boston, MA 02115, USA; 2 Department of Biostatistics, Harvard TH Chan School of Public Health, Boston, MA 02115, USA

**Keywords:** health, heat, vulnerability, built environment, public housing, indoor environmental quality, temperature

## Abstract

In the Northeastern U.S., future heatwaves will increase in frequency, duration, and intensity due to climate change. A great deal of the research about the health impacts from extreme heat has used ambient meteorological measurements, which can result in exposure misclassification because buildings alter indoor temperatures and ambient temperatures are not uniform across cities. To characterize indoor temperature exposures during an extreme heat event in buildings with and without central air conditioning (AC), personal monitoring was conducted with 51 (central AC, *n* = 24; non-central AC, *n* = 27) low-income senior residents of public housing in Cambridge, Massachusetts in 2015, to comprehensively assess indoor temperatures, sleep, and physiological outcomes of galvanic skin response (GSR) and heart rate (HR), along with daily surveys of adaptive behaviors and health symptoms. As expected, non-central AC units (T_mean_ = 25.6 °C) were significantly warmer than those with central AC (T_mean_ = 23.2 °C, *p* < 0.001). With higher indoor temperatures, sleep was more disrupted and GSR and HR both increased (*p* < 0.001). However, there were no changes in hydration behaviors between residents of different buildings over time and few moderate/several health symptoms were reported. This suggests both a lack of behavioral adaptation and thermal decompensation beginning, highlighting the need to improve building cooling strategies and heat education to low-income senior residents, especially in historically cooler climates.

## 1. Introduction

Extreme heat events are a significant public health threat that are increasing in frequency, duration, and severity with climate change [1,2]. Globally, people were exposed to an average of 1.4 more heatwave (HW) days in from 2000 to 2017 than from 1986 to 2005, and 18 million more extreme heat exposure events occurred in 2017 than in 2016 [3]. In Boston, Massachusetts (MA), there was an average of 11 days above 90 °F per year from 1971 to 2000, but it is expected to experience up to 40 of these hot days by the year 2030, and up to 90 hot days by 2070, depending on greenhouse gas emission trajectories [4].

As temperatures increase, the human body becomes less effective at thermoregulation, which has direct and indirect health impacts on cardiovascular, respiratory, renal, pancreatic, digestive, cerebrovascular, and cognitive functions that result in significant morbidity and mortality. Extreme heat events, such as the 2003 European HW and the 2015 HW in India, result in extraordinary loss of life [4,5]. However, temperatures that are currently designated as extreme will become more common with climate change, and are expected to result in significantly higher heat-related mortality and morbidity in the U.S. and throughout the world by the end of the century [6,7,8].

The impacts of extreme heat have the potential to widen existing disparities in economically disadvantaged and health-compromised populations in the U.S., as those who are most vulnerable will be most impacted by extreme heat with climate change. Human vulnerability to extreme heat arises from many risk factors, including, but not limited to, infants and the elderly, social isolation, low income, and low education [9]. Semenza et al. found that during the Chicago HW of 1995, there were higher odds of death for those with pre-existing medical conditions [10], and chronic disease has been found to be a risk factor for heat illness across the U.S. [11]. Pre-existing disease can alter sympathetic nervous system response, preventing cardiac responses that allow for thermoregulation adjustments during extreme heat exposure [12].

Older adults are more susceptible to adverse health outcomes during extreme heat events due to high prevalence of pre-existing disease, medications, and autonomic nervous system impairments affecting the thermoregulation and perception of extreme temperature exposure [13]. Skin-related vasoconstriction and vasodilation have both been found to be diminished in older adults, even when considering younger individuals at similar fitness and hydration levels [14]. The number of elderly individuals who are at least 65 years old in the U.S. is expected to more than double by mid-century, and will make up one-quarter of our population by 2060 [15], resulting in a larger group of susceptible individuals that will be vulnerable to the health impacts of HWs.

Air conditioning (AC) has been widely adopted as the main adaptation strategy to mitigate extreme heat exposures indoors and is extremely important in reducing the incidence of poor health outcomes during extreme heat [10,16]. Quinn et al. (2017) found that central AC is more effective as overcoming internal thermal loads during extreme heat and cooling indoor environments compared to portable or window AC units [17]. A 2011 study in New York City has found that, of seniors or adults in poor health, 34% surveyed did not own or use AC during extreme heat events, 30% were unaware of heat warnings, and many did not perceive themselves to be at risk of the extreme temperature exposures [18]. Between a fifth and a third of Massachusetts residents have central AC at home, while 20% of households lack any type of AC [19,20].

Despite the dependence on AC for cooling during extreme heat, one of the many protective factors often studied with regard to heat stress, the role of buildings is less well-understood. Depending on the building architecture, design, orientation, materials, and ventilation, extreme temperatures may be exacerbated indoors, as compared to outdoor temperatures during extreme heat events due to internal heat loads and the building’s thermal mass. Individuals who are most vulnerable to the health impacts of extreme heat are less likely to have access to or utilize AC. While upwards of 90% of residents in New York City were found to have some form of AC, roughly only 50% of public housing residents have access to AC [21]. Even in cities with widespread AC use, such as in Maricopa County, Arizona, nearly 40% of heat-related deaths happen indoors due to a variety of factors, including non-functioning ACs, lack of electricity, and disabling ACs to avoid high financial costs [22].

In the United States, adults can spend up to 90% of their time indoors [23]. In buildings without adequate cooling systems, people may be exposed to elevated indoor temperatures, thus increasing the risk of experiencing heat-related health effects. A great deal of the previous research about the health impacts resulting from extreme heat exposure has utilized ambient meteorological measurements to classify heat exposures, which can result in exposure misclassification in two ways: from buildings altering temperatures indoors where people are primarily exposed and from fine spatial differences in ambient temperatures that are not uniform across a city.

There are also a wide range of adaptive behaviors an individual can take that could mitigate or exacerbate heat exposure and poor health outcomes as a result. A past study in Detroit, Michigan found that behaviors like opening windows/doors, using fans or AC, and leaving the house have been used more than behaviors like changing clothes, showering, and going to the basement/porch/yard for urban-dwelling adults [24]. However, these behaviors are most frequently enacted in more moderate temperatures (23.8 °C–26.6 °C), and least used when indoor temperatures exceed 32.2 °C [24]. While there was a large majority (>90%) of older adults surveyed in Australia who reported wearing cooler clothing, closing curtains/shades, and drinking more fluids during hot days, less than 14% reported going to a cooler place [25]. During a 2013 HW in the UK, the elderly were the least likely to take similar protective measures against the heat, while those with higher income and education reported taking these measures often/always [26]. As is demonstrated here, the use of heat-mitigating adaptive behaviors varies widely.

Several of the risk factors associated to weakened thermoregulation during periods of heat stress are aggravated in older adults: lower capillary blood flow for radiative and convective cooling, lower sensitivity to thermal stimuli and core temperature-activated vasodilation, and maximal skin blood flow; all of these are detrimentally impacted by dehydration [27]. Drinking water during hot periods in urban areas has been found to be associated with a decreased risk of heatstroke [28]. Given the physiologic importance of vasodilation and its dependence on water intake, hydration is a key adaptive behavior that impacts human thermoregulation during periods of extreme heat.

In Cambridge, MA, the location of this study, buildings have been designed based on historical climate (i.e., colder winters and ocean-moderated milder summers). With warming summer temperatures, buildings without adequate cooling systems have the potential to retain heat, even during non-extreme events, in the future. This study characterized the indoor environmental quality (i.e., temperature, relative humidity, carbon dioxide, and noise) in the apartments of older adults residing in public housing in Cambridge, MA during an extreme heat event. In addition, personal physiological parameters were tracked with wearable devices, capturing daily adaptive behaviors, physiological responses to heat, and health symptoms. The use of affordable sensors and personalized monitoring provided a more comprehensive and representative documentation of participants’ exposures and experiences over the course of several summer days when an extreme heat event occurred compared to relying on ambient meteorological stations placed throughout a city. Improving the understanding of the exposures experienced by low-income seniors at home during extreme heat events has the potential to inform more equitable adaptation strategies that increase the resilience of this vulnerable population.

## 2. Materials and Methods

### 2.1. Study Design and Participants

A cohort of low-income seniors, living in public housing in Cambridge, MA, participated during the summer of 2015. This study took place over three periods: 29 June–3 July, 6 July–14 July, and 28 July–2 August, deploying study instruments when weather was predicted to be especially hot. Participants were recruited from two public housing units in Cambridge, MA. The first residence was a 180-unit multi-story high-rise building with five- and twelve-story sections that was originally built in 1973 and was renovated in 2013, and all residents had central AC (*n* = 24). The second residence was a 180-unit, cast-concrete 19-story high-rise building originally constructed in 1976 (non-central AC, *n* = 27) and had mixed-use AC with residents using either efficiently working window AC units, less efficiently working window AC units, or no window AC unit at all. In both buildings, residents received full energy subsidies to cover energy costs.

Prospective participants received details of the study objectives and protocols at informational meetings held at each of the study sites. Recruitment occurred on a rolling basis until recruitment targets (at least *n* = 20 for each building) were met; the research team only required that groups from both building types were balanced in terms of participant age and sex. Inclusion criteria required that the participant was at least 55 years of age, resided in either of the study buildings, and met a set of predetermined health conditions (not using oral or intravenous antibiotics or chemotherapy, was not using prednisone or nonsteroidal anti-inflammatory drugs, and did not currently have acute infectious disease (cold/flu, gastroenteritis, etc.)). There were no significant differences in the prevalence of pre-existing health conditions between participants living in these two buildings (Table 1). It was found that 92.6% and 79.2% of participants in the non-central AC and central AC groups reported that energy costs did not limit their use of AC (*p* = 0.88, Table 1), which is a common and important reason for not cooling a home environment in other low-income populations. Therefore, we assumed that these populations were comparable and living in either building was independent of the participant’s demographics, health status, energy financing restrictions, and potential health outcomes.

All subjects gave their informed consent for inclusion before they participated in the study. The study was conducted in accordance with the Declaration of Helsinki, and the protocol was approved by the Institutional Review Board (IRB) at the Harvard T.H. Chan School of Public Health (IRB15-1435).

### 2.2. Survey Instruments

Consented participants completed a baseline survey to assess their personal demographics (i.e., age, gender, height, weight, smoking, race, and ethnicity). The baseline survey also included information on sleep habits, as well as perception and satisfaction with existing indoor environmental quality (IEQ, i.e., thermal comfort, indoor air quality, acoustics, and lighting). Two key metrics assessed via the baseline survey were the number of pre-existing conditions each participant had been previously diagnosed with by a healthcare professional, and whether the participant had an appropriate heat action plan, defined by having at least one adaptive measure that they would do during an HW (turn on AC/fan, remove clothing, increase hydration, seek medical attention, etc.). The baseline survey is available in Table S1 (Supplementary Materials).

Participants were instructed to complete a self-administered survey every morning after waking for the duration of the study, which inquired about the previous day’s activities, IEQ, sleep quality and duration, and adaptive behaviors (i.e., beverage consumption, physical activity, and window opening/closing). We chose to also specifically assess hydration given its importance in cooling core body temperature via enhanced intracellular fluids to allow for enhanced vasodilation. Additionally, the other adaptive behavior questions (window opening/closing) were not consistently reported by study participants, while hydration (number of glasses of water) was consistent throughout the study period. We used the following symptom groups previously identified by the U.S. Environmental Protection Agency Building Assessment Survey and Evaluation (BASE) study as the most representative health outcomes associated to IEQ: neurocognitive (dizziness, nausea, headaches, and thirstiness); allergies (skin rash and sneezing); lower respiratory (coughing, breathing problems, and wheezing); irritation (nose bleeds, eye irritation, and sore throat); upper respiratory (ear pain, nasal drip, common cold, and sinusitis), mental health symptoms (tiredness, anxiety, irritation, and depression), and heat stress (nausea, numbness in hands/feet, dry skin, rash, sweating, and clammy skin) [29]. Musculoskeletal symptoms were not included in our survey as they were not a focus of this particular study. Instead, we expanded the number of symptoms associated with mental health disorders. Recognizing that neurocognitive health symptoms may also result from heat stress [30], even in young, healthy adult populations [31], these symptoms were combined with heat stress symptoms to assess symptoms experienced by the study participants. The daily survey is available in Table S2 (Supplementary Materials).

### 2.3. Environmental Measures

IEQ monitors (Netatmo, France) were installed in each participant’s bedroom to measure indoor dry-bulb temperature (°C), relative humidity (%), carbon dioxide concentration (CO_2_, ppm), and noise (dBa). The monitors were installed following a standardized protocol, ensuring they were away from sources of heat (computer screen, direct insolation, etc.) or drafts (e.g., windows and AC vents). Before deployment, CO_2_ was referenced to outdoor air (~400 ppm) to eliminate a drift error. CO_2_ drift and gain errors during deployment were estimated by collocating the IEQ monitors next to a recently calibrated instrument (Q-trak 7575, TSI Instruments, Shoreview, MN USA) inside a chamber, following ten stepwise increments from 400 to 3000 ppm. Values from the calibrated instrument were used as a reference to produce monitor-specific adjustment curves to match the experimentally derived values. Hourly outdoor weather variables were obtained from the local airport weather station (Logan International Airport, KBOS), located approximately five miles away from the study site. Indoor temperature exposures were analyzed as continuous measurements (5-min intervals) of daily means respective to each participant’s residential unit.

### 2.4. Physiologic Measures

Participants wore an actigraphy-based sleep tracker (Basis Peak watch, Intel, USA) on their non-dominant wrist and were instructed to wear it at all times, especially during sleep, and except when bathing/swimming. Tosses and turns during sleep were quantified by the tracker. The tracker used photoplethysmography to measure the heart rate (HR) in beats per minute (bpm), and galvanic skin response (GSR) in microsiemens at a 1-min resolution. Hourly HR and GSR means were utilized in statistical analyses.

### 2.5. Statistical Analyses

Building-level characteristics were analyzed with Mann–Whitney–Wilcoxon tests (IEQ variables), Student *t*-tests (demographic traits), binomial test of proportions (IEQ threshold comparisons), and paired *t*-tests (hydration). A binary variable having reported at least one moderate or severe heat- or non-heat-related health symptom in a given day was considered as a primary outcome. Generalized additive mixed models were used to evaluate the influence of mean hourly indoor temperatures and physiologic markers. GSR was log-transformed. To investigate the effect of the indoor temperatures on both heat- and non-heat-related self-reported health outcomes, we conducted a mixed-effects logistic regression model with a binomial distribution. Participant, nested within a building, was treated as a random effect in all models to account for repeated measurements within each subject and non-time-varying covariates, such as age, gender, U.S.-born status, and pre-existing health conditions, as well as other unquantifiable differences that may exist between buildings. Exploratory analyses for time spent outdoors and maximum daytime ambient temperature yielded few differences between or within individuals and groups, so these were excluded from the models. The final model evaluated the association of indoor maximum temperature with having at least one self-reported health outcome (binary) while controlling for that having at least two pre-existing conditions (binary) or having an appropriate heat action plan (binary). R (Version 3.5.0, R Core Team, Vienna, Austria) was used for all statistical analysis.

## 3. Results

### 3.1. Descriptive Results

There were no significant differences in demographic characteristics (age, gender, ethnicity, etc.) or in the prevalence of pre-existing health conditions between participants living in these two buildings (Table 1). The number of participants with a heat action plan was marginally different (*p* = 0.08) with more participants without central AC reporting protective measures. The average daily survey completion rates for the study were 79.0% for the central AC building and 77.5% for the non-central AC building throughout the study period. Therefore, we assumed that these populations were comparable and living in either building was independent of the participant’s demographics, health status, or potential health outcomes.

**Figure 1:**
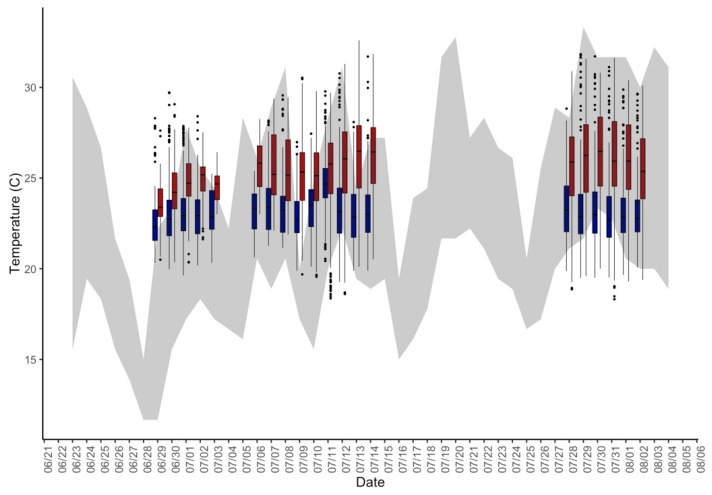
Indoor temperature distribution (boxplots) of non-central AC (red) and central AC (blue) groups; daily range of ambient temperature (grey shading).

**Figure 2:**
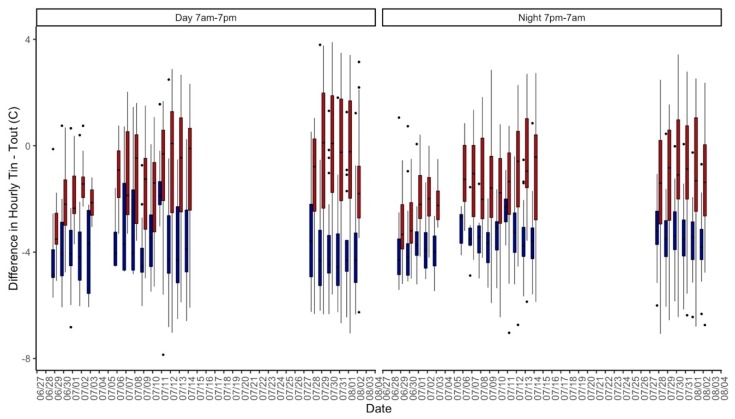
Difference between hourly indoor and outdoor temperatures (boxplots) of non-central AC (red) and central AC (blue) groups during both day (7 am–7 pm) and night (7 pm–7 am) periods.

As expected, mean indoor values of temperature, vapor pressure, noise, and CO_2_ were significantly higher in the non-central AC residences than in the central AC residences (*p* < 0.001). The mean indoor relative humidity was significantly higher in the central AC group than in the non-central AC building (*p* < 0.001), but absolute humidity was similar between building groups (*p* = 0.5356) (Table 1). Indoor temperatures of the non-central AC residences closely followed the ambient temperatures, but as outdoor temperatures rose, the indoor temperatures of the central AC residences dissociated from this correlation with ambient temperatures (Figure 1). In both the central AC and non-central AC buildings, the daytime differences between indoor and outdoor temperatures (AC: −3.7 °C, non-central AC: −1.1 °C) were significantly different than the night-time differences between indoor and outdoor temperature (AC: −3.7 °C, non-central AC: −1.5 °C) (*p* = 0.029 and *p* = 0.024 for daytime and night-time situations, respectively) (Figure 2). The difference in nighttime indoor-outdoor temperature difference between buildings was also statistically significant (*p* < 0.001). Even though mean indoor temperatures were cooler than ambient temperatures for both buildings, 45.7% (central AC) and 63.3% (non-central AC) of daytime hours and 64.5% (central AC) and 82.8% (non-central AC) of overnight hours had mean indoor temperatures that exceeded ambient temperatures, which were significantly different between buildings (*p* < 0.001).

### 3.2. Physiology and Sleep

**Figure 3:**
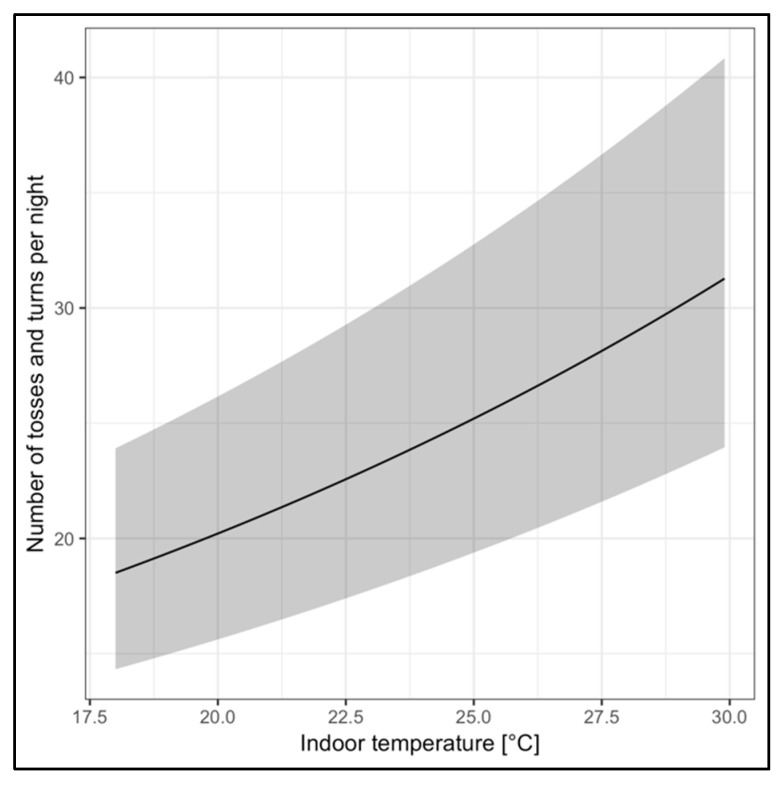
Relationship between indoor temperatures and number of tosses and turns during sleep periods. The gray shading represents the 95% confidence interval.

The relationship between tosses and turns and indoor temperature was modeled as a Poisson model of the toss and turn counts per night and viewed as a dependent variable, and mean temperature overnight and building were regarded as independent variables. When analyzing the reported duration of sleep from the daily surveys or the watches, there was no significant association with indoor temperature or building. However, the number of tosses and turns recorded by the watches during sleep, as quantified by the personal sensors, increased with indoor mean temperatures (Figure 3). No significant effect of building was observed (*p* = 0.496).

**Figure 4:**
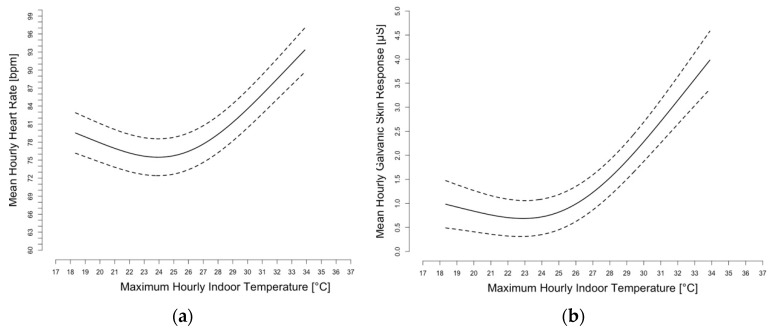
Non-linear association between hourly indoor temperatures and mean hourly heart rate (HR), after adjusting for building (**a**). Non-linear association between hourly indoor temperatures and mean hourly log-transformed galvanic skin response (GSR), after adjusting for building, and HR (**b**). The dotted lines represent the 95% confidence intervals.

The objective physiologic parameters, as measured by the watches, showed that the maximum hourly indoor temperature was a significant predictor (*p* < 0.001) of mean hourly HR after adjusting for building and had a non-linear relationship. An optimum of HR and GSR was found at around 24 °C (~75.2 °F). Both GSR and HR were found to increase once temperatures exceeded approximately this optimum threshold of 24 °C. (Figure 4). Both of these objective parameters demonstrated that as indoor temperatures were above or below this threshold, there were significant decrements in these physiologic markers. This threshold is higher than the threshold documented in a study using similar methodology for assessing the impact of heat on students’ cognitive functioning (~22 °C) [31].

### 3.3. Perception and Self-Reported Health Symptoms

**Figure 5:**
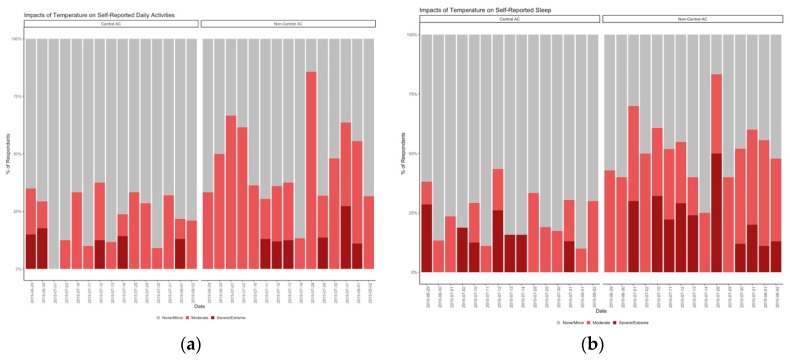
(**a**) Percent of respondents indicating the impact the thermal conditions of their apartment have on their self-reported daily activities. (**b**) Percent of respondents indicating the impact the thermal conditions of their apartment have on their self-reported sleep.

As expected, the number of participants who reported their unit was too hot increased as the study period went on, which follows increases in temperature (Figure S1, Supplementary Materials). The impact of thermal conditions at home on daily activities (Figure 5a) and on sleep (Figure 5b), as assessed through the daily survey, worsened throughout the study period. Approximately equal proportions of residents were satisfied, dissatisfied, or neither satisfied or dissatisfied between the non-central and central AC buildings at baseline (Figure S2, Supplementary Materials). However, almost 75% of participants in the non-central AC building indicated that their apartment’s temperature was too hot, while less than 25% of central AC building occupants felt too hot at baseline (Figure S2, Supplementary Materials).

Comparing hydration throughout the study period, the number of glasses of water consumed on hot days during the study (T_Max_ > 32.2 °C (90 °F)) was not significantly different than on days below this threshold for either building (central AC: *p* = 0.48; non-central AC: *p* = 0.64). Hydration was also not significantly different on days that were warm but did not meet extreme heat thresholds (>29.4 °C (85 °F)) (central AC: *p* = 0.89; non-central AC: *p* = 0.21). Thus, despite an enhanced perception of hotter indoor living conditions and reporting greater impact of these thermal conditions on routine living functions, the study participants did not drink more water, which many highlighted as a key action to take to protect themselves from heat stress.

During the study period, 9 participants reported 177 moderate and severe health symptoms, with 44 of those symptoms being heat-related. The number of daily self-reported health symptoms in any category, as well as those related to heat, was highest in the units at the highest indoor temperature quartiles for those with and without pre-existing conditions. The final models for experiencing at least one self-reported health outcome, which follows on previously methodology in Quinn and Shaman [32], controlled for mean daily indoor temperature and having either pre-existing conditions or a personal heat action plan. Given the relative rarity of these outcomes being reported in the study period, models examining the interaction between these covariates or with additional covariates were not assessed because of lack of statistical power (Table S3 and Table S4, Supplementary Materials; Equations (1) and (2)).

## 4. Discussion

Many studies have shown the association between heat exposure and health outcomes through the use of laboratory-controlled settings and/or through ambient exposure metrics. This study comprehensively characterized individual temperature exposures at home and IEQ using personal sensors and wearable devices. Simultaneously, it also incorporated environmental and behavioral factors that influence resilience and adaptive capacity during extreme heat events, which influence an individual’s susceptibility to poor health outcomes during an extreme heat event. The results showed that indoor temperatures alter physiology, increasing both GSR and HR, with an optimum range for these physiologic markers centered at around 24 °C. Simultaneously, participants’ perception of indoor temperatures and the impact of these exposures on their activities and sleep worsened. However, these changes did not result in a significant change in hydration, signaling a lack of enacting an important adaptive behavior and thermal decompensation beginning as the body is unable to thermoregulate.

While outdoor temperatures are associated with public health outcomes, these analyses underscore the importance of characterizing exposures to temperatures indoors and accounting for building conditions for understanding health risks during and after extreme heat events. All of these residents, given their close proximity, were exposed to the same ambient conditions, but their residential building played a role in either exacerbating or mitigating heat exposures. It also demonstrated the complex nature of heat vulnerability, pre-existing conditions, adaptive behaviors, and access to and use of AC (either well-functioning window units or less efficient window units for those in the non-central AC building). The success of using wearable sensors devices to monitor indoor and personal exposures demonstrated the feasibility of resolving a previous research limitation [27,28]. As sensor technology develops further and becomes more widespread, it may be possible to intervene before someone experiences life-threatening heat stress.

About 37% of all public housing residents in the U.S. are seniors over age 62, while 48% of public housing residents in Massachusetts are seniors [32]. By 2030, it is projected that 20% of the U.S. population will be seniors [15] and almost 90% of seniors have reported that they want to stay in their homes, living independently, as long as possible [33]. Living environments that are adequately cooled to meet the needs of older adults and effective personal heat mitigation strategies will be crucial to protect the health of seniors in the future in a warmer climate. Harnessing available technologies to track the IEQ of living environments, as well as the health conditions, of this population in real time, has the potential for greater independence while aging in place.

The suite of measurements utilized in this study revealed several interesting features of the indoor environments of public housing senior residents. Even though the majority of those without central AC did have access to window AC units, at a variety of efficiency and frequency of use preferences, these residences were continually warmer than those with central AC. This follows on findings from Quinn et al. (2017) that apartments cooled by window and portable AC are warmer than those with central AC and retain the heat for upwards of 1 day after ambient heat subsides [17]. In this study, window AC units were of a variety of ages and conditions, or were not used regularly or soon enough by the participants, yielding increasingly hotter temperatures indoors that persist as outdoor temperatures rise and then subside.

During the overnight hours, the differences between the indoor and outdoor temperatures were most pronounced across all units, and the building without central AC was significantly warmer than the building with central AC. Researchers have recently preferred a shift from the thermal comfort model where the percentage of satisfied occupants is based on climate chamber experiments to a thermal health model that involves not only environmental parameters but also individual physiological and psychological factors that are specific to vulnerable populations, like low-income seniors, to best protect public health [34,35,36].

Not being able to identify adaptive behaviors that would be protective during extreme heat events has been found to be a marker of increased susceptibility to heat exposure. Analyses suggested that having a heat action plan did increase the odds of reporting any or heat-related health symptoms, although not significantly. When assessing these results in the context of hydration, or the lack thereof, the result suggested that although residents were aware of protective actions during heat, they did not implement them in effective ways. This highlights an important area for intervention.

Takahashi et al. (2015) demonstrated that adaptive cooling behaviors were improved in elderly residents in Japan that received heat wave warnings in tandem with water distribution [37]. Although a great deal of the existing literature on the effectiveness of hydration during thermal stress has been done in the field of sports medicine and/or with adults under physical exertion and the study population here is largely sedentary, water levels within the bodies of older adults and thirst responses decrease with age [27], so thermoregulatory responses are weakened in these individuals. Marked changes in GSR as temperatures increased demonstrated that the study participants were losing water at a greater rate as it got hotter indoors, without increasing hydration to replenish body fluids. Future studies should examine hydration, in quantity and quality, in coordination with other adaptive behaviors for the most comprehensive description of adaptive responses.

These results provide evidence that not all vulnerability metrics, e.g., not having central AC, older age, low income, and pre-existing conditions, are equally determinant of poor health during extreme heat exposures. Åström et al. (2011) have noted the importance of studying non-fatal events and how housing modifies these outcomes in the elderly [38]. While the association between indoor temperatures, pre-existing conditions, and all health outcomes was not significant when modeled, the results of this study suggested areas of further investigation with a higher sample size, extended assessment periods, and more robust measurements of these outcomes.

While increasing utilization of AC is an obvious response to increasing heat, it will increase energy demand and shed more heat to ambient environments. AC should not be the only adaptation considered, in part because of the inherent economic disparities about who can afford to purchase AC and pay for the energy. Despite AC units becoming more efficient over time [39], utilizing AC with our present energy infrastructure contributes to positive feedback cycles that further propagate climate change and ambient air pollution emissions on hot days when electricity is generated by fossil fuels [40,41]. The exhaust heat from current air-cooling systems has also been found to exacerbate the problem of urban heat island effect [42]. Further, the refrigerants used in ACs, hydrofluorocarbons, are potent greenhouse gases that contribute to climate change. Thus, even though AC is effective in mitigating heat exposures indoors and protecting public health, additional adaptation strategies, such as adopting alternative refrigerants as proposed in the Kigali Amendment [43], designing our buildings and cities to be less thermally absorptive, and rapidly expanding renewable energy uptake, must be incorporated into our long-term solutions to reduce health-damaging air pollutants and greenhouse gas emissions that contribute to climate change, and to make climate adaptation more economically attainable for all sectors of the population, especially those who are most vulnerable.

Modeling studies have found that in the UK, external shutters, especially when combined with energy efficient upgrades to buildings, reduced heat-related mortality from 30% to 52% [44], while less heat-absorbent materials incorporated into buildings can significantly reduce indoor heat stress risk [45]. Energy-intensive AC will become more prevalent in homes across the world in coming decades and will be a crucial strategy in protecting populations from heat exposures. However, as discussed by Kwok and Rajkovich (2010), thermal comfort standards should be re-evaluated to better balance the mitigation of greenhouse gas emissions and provide healthful indoor environments for vulnerable populations to best equip our built environment for climate change [46]. Their point is underscored by the fact that current thermal comfort standards are not based on elderly populations with more underlying health risks.

Alternative adaptive/coping strategies need to be available for a growing number of vulnerable people in case cooling is not always available, like for example, during a power outage. Designing for the passive habitability of buildings, which would allow “habitable indoor conditions without power for limited amounts of time” [47,48] creates greater resilience among our building stock and better protects the individuals inside.

While the findings of this research present valuable information for public health practitioners and building managers, there were several limitations that are important to recognize. There were short gaps in the study period due to weather forecasts predicting only 1–2 days of extreme heat in mid-July when our objective was to characterize a longer period of extreme heat and its impact on indoor environments, and therefore the instruments were only deployed when weather was predicted to be especially hot. It would have been preferable to have a clearer HW signal to create more delineated ambient exposure periods with lower temperatures during the other study days, but unfortunately this was not possible with this type of study outside of a laboratory setting. However, the magnitude of these results may increase given even more extreme ambient temperature conditions.

The small sample size (*n* = 51) and rarity of the reporting of moderate/severe health symptoms (only 9 participants) may present limitations and limit our analysis of the impact of indoor heat on health symptoms in this study. Additionally, the daily surveys were self-administered at home, which is less controlled than if they were recorded by the study team. Even though we lacked information on the functionality and efficiency of the window AC units that were present, personal questions about thermal conditions of the apartments and participant activities were corroborated with environmental and personal monitors that recorded indoor temperatures, which reinforce the reliability of these measurements. Participants used time-activity logs but their data on hourly location was not very complete across the study period. Given the associations found between indoor temperature exposure and sleep and physiology, as well as with poor health symptoms, it is plausible to assess whether or not sleep is a mediating factor between indoor temperatures and poor health symptoms. We were unable to assess this here, due to the rarity of health symptoms reported, but are eager to explore in future studies.

The amount of time indoor and outdoor was consistent (about 2–3 hours/day outdoors) between all participants, regardless of building, as well as over time, so indoor temperatures were used as the exposure of interest since people were spending the majority of their time indoors and at home. Even though outdoor temperature exposures are not characterized at the individual level, the vast amount of indoor temperature exposure data utilized decreases the amount of exposure misclassification that may exist in other studies. However, some exposure misclassification may still exist if participants were outside or not at home for the entirety of the day

The limited, older age range of the participants may mean that the results are not generalizable to younger, healthier populations who may be less susceptible to extreme heat exposures. However, given the severity of climate change and future extreme heat scenarios, compounded by an aging population in the United States, there are likely still important lessons to be learned from this analysis for an increasing proportion of our population. Further research should examine the influence of the building in modifying health outcome and physiologic measures in other demographic populations of interest, as well as to other geographic locations, potentially over a longer period of times to account for multiple and recurring extreme heat events.

## 5. Conclusions

In this study, sensors were used to quantify indoor temperatures during an extreme heat event, reducing temperature exposure misclassification, and associated increases in HR and GSR of 51 older adults living in public housing. Assessment of adaptive behaviors demonstrated that hydration did not increase and the reporting of subclinical health symptoms was rare during the same time period. This signals that despite thermal decomposition occurring, even during a heat event that was not severe, older adults are not effectively implementing adaptive behaviors, like drinking more fluids.

With warming summer temperatures, buildings without adequate cooling systems have the potential to retain heat, even during non-extreme events, and individuals who are most vulnerable to the health impacts of extreme heat are less likely to have access to or utilize AC. Understanding how building elements modify indoor temperatures and monitoring indoor temperature conditions in real time is essential knowledge for identifying vulnerable indoor environments and informing adaptation and mitigation strategies to reduce the impact of excessive heat. Strategies that do not solely depend on AC, like enhanced shading/shutters and less heat-absorbed materials, will be vital adaptation solutions to buildings that maintain elevated indoor temperatures during extreme heat. Further, local heat action plans and HW programing by public health practitioners should account for the indoor temperature exposures that may be exacerbated by the built environment to improve messaging, public health campaigns, and distribution of cooling resources (hydration, transportation to cooling shelters, energy subsidies for AC, etc.).

## Figures and Tables

**Table 1:** Descriptive statistics for demographics, pre-existing medical diagnoses, and indoor environmental quality of study participants living in public housing with and without central air conditioning (AC) in Cambridge, MA.

Descriptive Statistics	Non-central AC (*n* = 27)	Central AC (*n* = 24)	*p*-Value
Demographic information			
Age, mean (SD) years	65.3 (7.9)	65.5 (7.5)	0.90
Sex, *n* (%) male	11 (45.8)	11 (40.7)	0.71
Race, *n* (%) non-white	7 (29.2)	10 (37.0)	0.83
Born in the United States, *n* (%)	16 (66.7)	21 (77.8)	0.37
Good + self-assessment of health, *n* (%)	19 (70.4)	13 (54.1)	0.23
Ever smoker, *n* (%)	15 (62.5)	21 (77.8)	0.23
Energy costs (do not limit AC use), *n* (%)	25 (92.6)	19 (79.2)	0.88
Have a heat action plan, *n* (%)	20 (74.1)	13 (54.2)	0.08
Indoor environmental quality			
Temperature, mean (SD) (°C)	25.6 (2.28)	23.2 (1.8)	<0.001
Relative humidity, mean (SD) (%)	57.9 (7.3)	67.0 (6.7)	<0.001
Absolute humidity, mean (SD) (g/m^3^)	0.0140 (0.002)	0.0141 (0.001)	0.5356
Vapor pressure, mean (SD) (hPa)	1939.7 (343.9)	1935.9 (209.0)	<0.001
Noise, mean (SD) (dB)	54.3 (8.1)	48.0 (8.1)	<0.001
Carbon dioxide, mean (SD) (ppm)	559 (176.9)	546 (161.2)	<0.001
Pre-existing medical diagnosis, *n* (%)			
Chronic migraines	7 (25.9)	7 (29.2)	0.80
Severe headaches	5 (19.2)	7 (29.2)	0.41
Asthma	8 (30.8)	3 (12.5)	0.12
Chronic bronchitis	6 (23.1)	5 (20.8)	0.85
Allergies	12 (44.4)	12 (50.0)	0.69
Eczema	3 (11.1)	3 (12.5)	0.88
Hives	2 (7.7)	3 (12.5)	0.57
Sleep apnea	9 (34.6)	8 (33.3)	0.92
ADD/ADHD	3 (11.1)	5 (20.8)	0.34
Hearing loss	5 (18.5)	4 (16.7)	0.86
Thyroid	5 (18.5)	4 (16.7)	0.86
Diabetes	9 (33.3)	7 (29.2)	0.75
Heart disease	5 (16.7)	4 (16.7)	0.81
Chronic fatigue/Fibromyalgia	2 (7.4)	4 (16.7)	0.31
Depression	7 (25.9)	10 (41.7)	0.23
Anxiety	8 (29.6)	12 (50.0)	0.14
COPD	4 (14.8)	2 (8.3)	0.74

## Supplementary Materials

The following are available online at https://www.mdpi.com/1660-4601/16/13/2373/s1, Figure S1: Satisfaction and perception of indoor thermal conditions at the baseline of the study period, Figure S2: Percent of respondents indicating their perception of the temperature of their apartment during the study period. Table S1: Baseline survey used to assess personal demographics, sleeping habits, perception/satisfaction with IEQ, and pre-existing demographics, Table S2: Daily survey used to assess the previous day’s activities, IEQ, sleep quality, adaptive behaviors, health symptoms, Table S3: Generalized logistic mixed effect regression output Odds Ratios (OR) for any health symptom or heat-related health symptoms adjusting for daily mean indoor temperature and having at least 2 preexisting conditions (model 1) or and having a personal heat action plan (model 2), with participant as a random effect, Table S4: Generalized logistic mixed effect regression models.
