# Supplementary material for: Building Vulnerability in a Changing Climate: Indoor Temperature Exposures and Health Outcomes in Older Adults Living in Public Housing during an Extreme Heat Event in Cambridge, MA
Source: Int J Environ Res Public Health. 2019 Jul 4;16(13):2373. doi: 10.3390/ijerph16132373 (PMC6651653; doi:10.3390/ijerph16132373)
Supplement: Supplementary file 1 [file ijerph-16-02373-s001.pdf]

International Journal of
Environmental Research and Public Health

MDPI

Supplementary

Building Vulnerability in a Changing Climate: Indoor Temperature Exposures and Health Symptoms in Older Adults Living in Public Housing during an Extreme Heat Event in Cambridge, MA

Augusta A. Williams, John D. Spengler, Paul Catalano, Joseph G. Allen and Jose G. Cedeno-Laurent

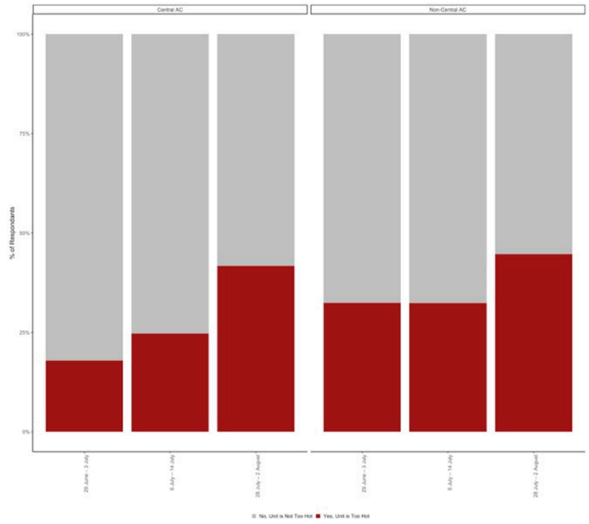

Figure S1. Percent of respondents indicating the thermal perception of their apartment during the 3 periods of the study.

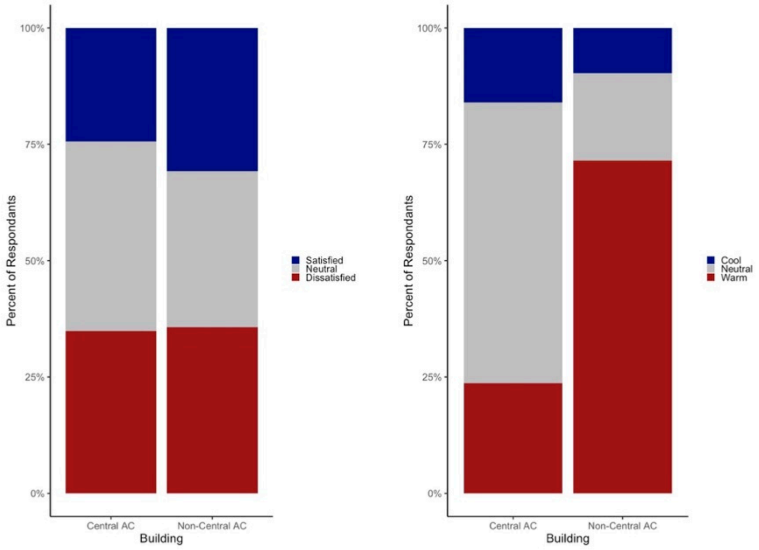

(**a**) (**b**)

Figure S2. Percent of respondents indicating the satisfaction with (**a**) and perception of (**b**) the thermal conditions of their apartment at the baseline of the study period.

Table S1. Baseline survey used to assess personal demographics, sleeping habits, perception/satisfaction with IEQ, and pre-existing demographics.

1. PID: Participant ID Number	
2. Building: Housing Development	
	1-Central AC
	2-NonCentral AC / Window AC
3. Date	
4. How many years have you lived here?	
5. How many people, beside you, are currently living in your apartment?	
6. How many people (total) are currently living in your apartment?	
7. Thinking about the last week, how would you describe the temperature in your bedroom?	
	1-Too cold
	2-Cold
	3-Slightly cold
	4-Neutral
	5-Slightly warm
	6-Hot
	7-Too warm
8. Thinking about the last week, please indicate the impact of temperature in your apartment on your activities:	
a. Daily Activities (e.g. writing, reading, chores, etc.)	
	1-No impact
	2-Minor impact
	3-Moderate impact
	4-Severe impact
	5-Extreme impact
b. Relaxing (e.g. watching tv)	
	1-No impact
	2-Minor impact
	3-Moderate impact
	4-Severe impact
	5-Extreme impact
c. Sleeping	
	1-No impact
	2-Minor impact
	3-Moderate impact
	4-Severe impact
	5-Extreme impact
9. Thinking about the last week, how many days did you take the following actions to modify the temperature in your apartment? Open/close the windows	
	1-Never
	2-1-2 days
	3-3-4 days
	4-5-6 days
	5-everyday
10. Thinking about the last week, how many days did you take the following actions to modify the temperature in your apartment? Open/close doors	
	1-Never
	2-1-2 days
	3-3-4 days
	4-5-6 days
	5-everyday

11. Thinking about the last week, how many days did you take the following actions to modify the temperature in your apartment? Adjust temperature controls using a fan	
	1-Never
	2-1-2 days
	3-3-4 days
	4-5-6 days
	5-everyday
12. Thinking about the last week, how many days did you take the following actions to modify the temperature in your apartment? Adjust temperature controls using the air conditioner	
	1-Never
	2-1-2 days
	3-3-4 days
	4-5-6 days
	5-everyday
13. Thinking about the last week, how many days did you take the following actions to modify the temperature in your apartment? Other	
	1-Never
	2-1-2 days
	3-3-4 days
	4-5-6 days
	5-everyday
14. Are the temperature controls in your apartment easy to access?	
	1-Yes
	2-No
	3-NA
15. Overall, how satisfied have you been with the temperature in your apartment during the last week?	
	1-very satisfied
	2-satisfied
	4-neutral
	6-dissatisfied
	7-very dissatisfied
16. Overall, how satisfied were you with the temperature in your apartment during this past winter?	
	1-very satisfied
	2-satisfied
	4-neutral
	6-dissatisfied
	7-very dissatisfied
17. What do you do when your apartment gets too hot in the summer? (check all that apply)	
	a. Shed clothes
	b. Adjust shades
	c. Adjust windows
	d. Use AC
	e. Use a fan
	f. Use a ceiling fan
	g. Adjust the thermostat
	h. Other___________
18. What do you do when your apartment gets too hot in the winter? (check all that apply)	
	a. Shed clothes
	b. Adjust shades
	c. Adjust windows
	d. Use AC
	e. Use a fan
	f. Use a ceiling fan
	g. Adjust the thermostat
	h. Other___________

19. What do you do when your apartment gets too cold in the summer? (check all that apply)	
	a. Shed clothes
	b. Adjust shades
	c. Adjust windows
	d. Use AC
	e. Use a fan
	f. Use a ceiling fan
	g. Adjust the thermostat
	h. Other___________
20. What do you do when your apartment gets too cold in the winter? (check all that apply)	
	a. Shed clothes
	b. Adjust shades
	c. Adjust windows
	d. Use AC
	e. Use a fan
	f. Use a ceiling fan
	g. Adjust the thermostat
	h. Other___________
21. What is the normal thermostat temperature in your apartment in the summer?	
22. What is the normal thermostat temperature in your apartment in the winter?	
23. Does the temperature of your apartment generally stay constant throughout the day and night?	
	1-yes
	2-no
a. If you answered No to the previous question, what do you think are the reasons for the temperature change throughout the day and night?	
	a. Thermostat doesn't work properly
	b. Temperature outside is too hot or cold
	c. The sun heats up the apartment interior
	d. Other ________________
24. Overall, how satisfied are you with the temperature in your apartment currently?	
	1-very satisfied
	2-satisfied
	4-neutral
	6-dissatisfied
	7-very dissatisfied
25. What would you guess is the temperature of this room right now?	
26. Thinking about the past week, express your satisfaction with the following indoor air quality parameters in your apartment:	
a. Air drafts	
	1-very dissatisfied
	2-dissatisfied
	4-neutral
	6-satisfied
	7-very satisfied
b. Air freshness	
	1-very dissatisfied
	2-dissatisfied
	4-neutral
	6-satisfied
	7-very satisfied
c. Odor intensity	
	1-very dissatisfied
	2-dissatisfied
	4-neutral
	6-satisfied

	7-very satisfied
27. Thinking about the last week, please indicate the impact of indoor air quality in your apartment on your activities:	
a. Daily Activities (e.g. writing, reading, chores, etc.)	
	1-No impact
	2-Minor impact
	3-Moderate impact
	4-Severe impact
	5-Extreme impact
b. Relaxing (e.g. watching tv)	
	1-No impact
	2-Minor impact
	3-Moderate impact
	4-Severe impact
	5-Extreme impact
c. Sleeping	
	1-No impact
	2-Minor impact
	3-Moderate impact
	4-Severe impact
	5-Extreme impact
28. Thinking about the last week, how many days did you take the following actions to modify the indoor air quality in your bedroom?	
a. Open/close windows	
	1-Never
	2-1-2 days
	3-3-4 days
	4-5-6 days
	5-everyday
b. Open/close doors	
	1-Never
	2-1-2 days
	3-3-4 days
	4-5-6 days
	5-everyday
c. Use air freshener	
	1-Never
	2-1-2 days
	3-3-4 days
	4-5-6 days
	5-everyday
d. Use incense	
	1-Never
	2-1-2 days
	3-3-4 days
	4-5-6 days
	5-everyday
e. Other ______________________	
	1-Never
	2-1-2 days
	3-3-4 days
	4-5-6 days
	5-everyday
29. Overall, how satisfied have you been with the indoor quality inside your apartment during the last week?	

	1-very dissatisfied
	2-dissatisfied
	3-neutral
	4-satisfied
	5-very satisfied
30. Thinking about the last week, express your satisfaction with the following acoustic conditions in your apartment.	
a. Noise from the outside	
	1-very dissatisfied
	2-dissatisfied
	4-neutral
	6-satisfied
	7-very satisfied
b. Noise from other rooms within the building	
	1-very dissatisfied
	2-dissatisfied
	4-neutral
	6-satisfied
	7-very satisfied
c. Noise from the lights/heat/plumbing/ventilation system	
	1-very dissatisfied
	2-dissatisfied
	4-neutral
	6-satisfied
	7-very satisfied
31. Thinking about the last week, please indicate the impact of acoustic conditions in your apartment on your activities.	
a. Daily Activities (e.g. writing, reading, chores, etc.)	
	1-No impact
	2-Minor impact
	3-Moderate impact
	4-Severe impact
	5-Extreme impact
b. Relaxing (e.g. watching tv)	
	1-No impact
	2-Minor impact
	3-Moderate impact
	4-Severe impact
	5-Extreme impact
c. Sleeping	
	1-No impact
	2-Minor impact
	3-Moderate impact
	4-Severe impact
	5-Extreme impact
32. Overall, how satisfied have you been with the amount of noise inside your apartment during the last week?	
	1-very dissatisfied
	2-dissatisfied
	4-neutral
	6-satisfied
	7-very satisfied
33. Thinking about the last week, express your satisfaction with the following lighting conditions in your apartment.	

a. Amount of daylight	
	14-very dissatisfied
	15-dissatisfied
	17-neutral
	19-satisfied
	20-very satisfied
b. Amount of electric lighting	
	14-very dissatisfied
	15-dissatisfied
	17-neutral
	19-satisfied
	20-very satisfied
34. Thinking about the last week, have you experienced any of the following lighting conditions in your bedroom?	
a. Frequent flickering lighting	
	1-yes
	2-no
b. Electric lighting with an undesirable color	
	1-yes
	2-no
c. Insufficient desk lighting	
	1-yes
	2-no
d. Glare on the television	
	1-yes
	2-no
e. Excess light coming from outdoors while trying to sleep	
	1-yes
	2-no
f. Too bright	
	1-yes
	2-no
g. Too dark	
	1-yes
	2-no
35. Thinking about the last week, please indicate the impact of lighting conditions in your apartment on your activities.	
a. Daily Activities (e.g. writing, reading, chores, etc.)	
	1-No impact
	2-Minor impact
	3-Moderate impact
	4-Severe impact
	5-Extreme impact
b. Relaxing (e.g. watching tv)	
	1-No impact
	2-Minor impact
	3-Moderate impact
	4-Severe impact
	5-Extreme impact
c. Sleeping	
	1-No impact
	2-Minor impact
	3-Moderate impact
	4-Severe impact

	5-Extreme impact
36. Overall, how satisfied have you been with the lighting conditions in your apartment during the last week?	
	1-very dissatisfied
	2-dissatisfied
	4-neutral
	6-satisfied
	7-very satisfied
37. Thinking about the last week, how would you describe your sleep? (Choose all that apply)	
a. I have difficulties getting to sleep	
	1-always
	2-sometimes
	3-never
b. I wake up in the middle of the night	
	1-always
	2-sometimes
	3-never
c. I have difficulties waking up	
	1-always
	2-sometimes
	3-never
d. I can barely stay awake	
	1-always
	2-sometimes
	3-never
e. feel refreshed when I wake up	
	1-always
	2-sometimes
	3-never
f. I take medications to help me sleep	
	1-always
	2-sometimes
	3-never
g. I wear an eyemask to sleep	
	1-always
	2-sometimes
	3-never
h. I wear earplugs to sleep	
	1-always
	2-sometimes
	3-never
i. I use a sound machine.	
	1-always
	2-sometimes
	3-never
j. Other___________	
	1-always
	2-sometimes
	3-never
38. On average, how many hours (a day) have you slept during the last week?	
	1-4 hours or less
	2-5 hours
	3-6 hours
	4-7 hours
	5-8 hours
	6-9 hours or more

39. Has a health professional ever told you that you had any of the following conditions?	
1-yes	
2-no	
	Migraines
	Severe Headaches
	Asthma
	Chronic Bronchitis/Post Nasal Drip
	Chronic Runny Nose
	Chronic Sinus Infection
	Other Lung Conditions___
	Allergies
	Psoriasis
	Eczema
	Hives
	Sleep Apnea/Sleep Disorder
	Immune Disorder
	ADD/ADHD
	Hearing Condition
	Raynaud's Syndrome Or Problems With Circulation In Your Extremities
	Thyroid Condition
	Diabetes
	Heart Disease
	Chronic Fatigue/Fibromyalgia
	Depression
	Anxiety
	Other Major Chronic Disease________________________
40. Thinking about the past week, how many days have you experiences the following symptoms? (Check all that apply)	
1-never	
2-1-2 days	
3-3-4 days	
4-5-6 days	
5-everyday	
	Difficulty Remembering Things Or Concentrating
	Unusual Tiredness, Fatigue, Or Drowsiness
	Tension, Irritability, Or Nervousness
	Feeling Depressed
	Dizziness Or Lightheadedness
	Headache
	Tired Or Strained Eyes
	Dry, Itching, Or Irritated Eyes
	Wheezing
	Sore Or Dry Throat
	Chest Tightness
	Stuffy Or Runny Nose, Or Sinus Congestion
	Pain Or Arthritis In Back, Shoulder, Or Neck
	Cough
	Sneezing
	Shortness Of Breath
	Nausea Or Upset Stomach
	Dry Or Itchy Skin
	Numbness In Hands Or Wrists
	Abnormal Rash
	Profuse Sweating

	Clammy Skin Or Chills
	Hallucinations
41. In the past 30 days, of those symptoms you have experienced from the previous page, indicate the correct treatment and impact of each below.	
a. Have you sought treatment? Are you currently being treated?	
	1-Have previously sought treatment
	2-Currently being treated
	3-Have previously sought treatment and currently being treated
b. Indicate the correct impact of the symptom below.	
	1-no impact
	2-mild impact
	3-severe impact
42. In general, would you say your health is…	
	1-excellent
	2-very good
	3-good
	4-fair
	5-poor
43. Have you smoked at least 100 cigarettes in your entire life?	
	1-yes
	2-no
44. Do you now smoke cigarettes every day, some days, or not at all?	
	1-everyday
	2-some days
	3-not at all
45. How often in the last 12 months have you seen signs of pests in your apartment? (This includes actually seeing pests and indications of pests such as feces and droppings, chewed packages, etc.)	
a. Roaches	
	1-never
	2-few times a year
	3-few times a month
	4-few times a week
	5-everyday
b. Mice	
	1-never
	2-few times a year
	3-few times a month
	4-few times a week
	5-everyday
c. Other__________	
	1-never
	2-few times a year
	3-few times a month
	4-few times a week
	5-everyday
46. Have you seen any mold on any surfaces (ie. walls, ceilings, floors in your apartment in the past year (12 months)? (Mold is a growth of fungi on a surface. In an apartment, it usually appears as a dark patch of 'fuzzy' growth on a wall, ceiling or other surface that has been wet for a period of time.) **very small mold patches in or around bathroom tiles should not be included.	
	4-yes
	5-no

	6-don't know
47. Do you currently have mold in your apartment?	
	1-yes
	2-no
	3-don't know
48. When the temperatures are really hot, what are some ways that you receive help?	
49. How often do you attend social events in the community?	
	1-never
	2-few times per year
	3-few times per month
	4-few times a week
	5-everyday
50. Do energy costs limit your use of an air conditioner?	
	1-yes
	2-no
	3-don't know
51. Do you have an air conditioner?	
	1-yes
	2-no
52. Overall, how satisfied have you been living in the apartment you currently live in?	
	1-very dissatisfied
	2-dissatsified
	4-neutral
	6-satisfied
	7-very satisfied
53. Age: how old are you, in years?	
54. Sex: What is your sex?	
	1 - female
	2-male
55. What is your height?	
56. What is your weight?	
57. Do you use hearing aids?	
	1-yes
	2-no
58. Do you use contact lenses?	
	1-yes
	2-no
59. Do you have continence issues or take any medications (like diuretics) that would influence water loss?	
	1-yes
	2-no
60. do you have pets?	
	1-yes
	2-no
a. yes, does that pet sleep in the same bed as you?	
	1-yes
	2-no
61. Please specifiy your ethnicity	
	1-White/Caucasian
	2-Black or African American
	3-Asian
	4-Native Hawaiian and other Pacific Islander
	5-American Indian or Alaska Native
	6-Latino

	7-Multiracial
	8-no response
	9-other_______________
62. Were you born in the United States?	
	1-yes
	2-no
63. Please share with us any other comments about your residential space that you think need improvement	

Table S2. Daily survey used to assess the previous day's activities, IEQ, sleep quality, adaptive behaviors, health symptoms.

1. Date
2. Participant Identification Number
3. Time survey was completed by the participant
4. Please indicate how many hours you spent in your living room yesterday.
5. Time Activity Log: Please check off which location you were in during each time period

	6am-9am	9am-12pm	12pm-2pm	2pm-4pm	4pm-6pm	6pm-8pm	8pm-10pm	10pm-12am	12am-6am
Living Room									
Bedroom									
Common Area									
Outside									
Other									

6. Did you experience any of the following environmental conditions while in your apartment yesterday? Please mark the box indicating your response.
a. Temperature too hot
b. Temperature too cold
c. Too loud
d. Too quiet
e. Too much air movement
f. Too little air movement
g. Air too humid
h. Air too dry
i. Unpleasant chemical odors
j. Other unpleasant odors (e.g. body odor, food odor, perfume)
7. Please indicate any negative impacts of temperature in your apartment on your activities yesterday. Please mark the box indicating your response.
a. Daily Activities (e.g. writing, reading, chores)
1-No Impact
2-Minor Impact
3-Moderate Impact
4-Severe Impact
5-Extreme Impact
b. Relaxing (e.g. watching tv)
1-No Impact
2-Minor Impact
3-Moderate Impact
4-Severe Impact
5-Extreme Impact
c. Sleeping
1-No Impact
2-Minor Impact
3-Moderate Impact
4-Severe Impact
5-Extreme Impact
8. Please indicate any negative impacts of noise in your apartment on your activities yesterday. Please mark the box indicating your response.
a. Daily Activities (e.g. writing, reading, chores)

1-No Impact
2-Minor Impact
3-Moderate Impact
4-Severe Impact
5-Extreme Impact
b. Relaxing (e.g. watching tv)
1-No Impact
2-Minor Impact
3-Moderate Impact
4-Severe Impact
5-Extreme Impact
c. Sleeping
1-No Impact
2-Minor Impact
3-Moderate Impact
4-Severe Impact
5-Extreme Impact
9. What would you guess is the temperature in your apartment right now?
10. How thirsty did you feel yesterday, on average?
1-not at all thirsty
2-a little thirsty
3-moderately thirsty
4-quite thirsty
5-extremely thirsty
11. How many full glasses of water (~8oz) did you have yesterday?
12. What time was your last drink of water yesterday?
1-6am-12pm
2-12pm-6pm
3-6pm-9pm
4-9pm-12am
5-12am-6am
13. How many drinks containing alcohol did you have yesterday?
14. What time was your last alcoholic drink yesterday?
1-6am-12pm
2-12pm-6pm
3-6pm-9pm
4-9pm-12am
5-12am-6am
15. How many caffeinated drinks did you have yesterday?
16. What times was your last caffeinated drink yesterday?
1-6am-12pm
2-12pm-6pm
3-6pm-9pm
4-9pm-12am
5-12am-6am
17. How much time did you spend watching TV or using a computer after 5pm yesterday before going to sleep?
18. How much time did you spend exercising yesterday?
19. How many times did you open or close your windows to modify the temperature in your bedroom?
1-0times
2-1 time
3-2+ times
20. How many times did you adjust the temperature controls to modify the temperature in your bedroom?
1-0times
2-1 time
3-2+ times

21. Did you take OTC or prescription medications to help you sleep yesterday before going to sleep:
1-Yes
2-No
22. What time was sleep medication 1 taken?
1-6am-12pm
2-12pm-6pm
3-6pm-9pm
4-9pm-12am
5-12am-6am
23. What time was sleep medication 2 taken?
1-6am-12pm
2-12pm-6pm
3-6pm-9pm
4-9pm-12am
5-12am-6am
24. What time was sleep medication 3 taken?
1-6am-12pm
2-12pm-6pm
3-6pm-9pm
4-9pm-12am
5-12am-6am
25. How many hours of sleep did you get last night?
26. What time did you go to sleep last night?
27. What time did you wake up this morning?
28. How long did it take you to fall asleep last night?
1-0-10 minutes
2-10-30 minutes
3-30-60 minutes
4- 60+ minutes
29. Was your sleep interrupted last night, and if so, how many times?
1-0 interruptions
2-1 interruption
3-2 interruption
4-more than 2 interruptions
30. How would you rate the quality of your sleep?
1-Very poor
2-Poor
3-Fair
4-Good
5-Very good
31. How rested or refreshed did you feel after you woke up?
1-Not at all rested
2-Slightly rested
3-Somewhat rested
4-Well-rested
5-Very well-rested
32. How happy did you feel over the course of the day yesterday?
14-Very unhappy
15-Unhappy
16-Neither happy nor unhappy
17-Happy
18-Very happy
33. How stressed did you feel over the course of the day yesterday?
4-Very Stressed
9-Stressed
5-Neither stressed nor relaxed

6-Relaxed
7-Very relaxed
34. Did anything unusual happen yesterday that you think affected your sleep last night?
35. Did you experience each of the following symptoms yesterday? If you answer yes, please indicate the severity of that symptom.
1-Mild Severity
2-Moderate Severity
3-Severe Severity
Difficulty remembering things or concentrating
Unusual tiredness, fatigue, or drowsiness
Tension, irritability, or nervousness
Feeling depressed
Dizziness or lightheadedness
Headache
Tired or strained eyes
Dry, itching, or irritated eyes
Wheezing
Sore or dry throat
Chest tightness
Stuffy or runny nose, or sinus congestion
Pain or arthritis in back, shoulder, or neck
Cough
Sneezing
Shortness of breath
Nausea or upset stomach
Dry or itchy skin
Numbness in hands or wrists
Abnormal rash
Profuse sweating
Clammy skin or chills
Hallucinations

Table S3. Generalized logistic mixed effect regression output Odds Ratios (OR) for any health symptom or heat-related health symptoms adjusting for daily mean indoor temperature and having at least 2 preexisting conditions (model 1) or and having a personal heat action plan (model 2), with participant as a random effect.

	**Model 1**		**Model 2**	
Any Health Symptom	OR	95% CI	OR	95% CI
Daily Mean Indoor Temperature (°C)	1.077	(0.944, 1.23)	1.009	(0.788, 1.29)
Preexisting Conditions	0.638	(0.255, 1.59)		
Heat Action Plan			1.556	(0.541, 4.47)
Heat-Related Health Symptoms	OR	95% CI	OR	95% CI
Daily Mean Indoor Temperature (°C)	1.032	(0.883, 1.205)	1.004	(0.812, 1.24)
Preexisting Conditions	0.273	(0.087, 0.854)		
Heat Action Plan			1.614	(0.601, 4.34)

Model 1 demonstrates a generalized logistic mixed effect regression for any health symptom or heat-related health symptoms adjusting for daily mean indoor temperature and having at least 2 preexisting conditions, with participant as a random effect (Equation (1)). Model 2 was a generalized logistic mixed effect regression for any health symptom or heat-related health symptoms adjusting for daily mean indoor temperature and having a personal heat action plan, with participant as a random effect (Equation (2)). The model results in Table 2 demonstrate that the odds of reporting any health symptom or a heat-related health symptom increased 7% and 3% times for each degree °C increase of daily mean indoor temperature on average, respectively. However, the 95% confidence intervals of these estimates contain the null (OR = 1). The odds of reporting a heat-related health symptom decreased by 72% times when having at least two preexisting conditions (95% CI = (0.09, 0.87). Given the acute nature of the symptoms assessed in this study, models using the same-day daily mean indoor temperature were utilized and shown here.

Table S4. Generalized logistic mixed effect regression models.

$$Logit\left(Health\ Symptoms_{ij} \middle| b_{1i}\right) = \beta_1 + \beta_2 * Daily\ Mean\ Indoor\ Temperature_{ij} + \beta_2 * At\ Least\ 2\ Preexisting\ Conditions + b1\left(Building(participant)\right) + \varepsilon_{ij} \quad (1)$$

$$Logit\left(Health\ Symptoms_{ij} \middle| b_{1i}\right) = \beta_1 + \beta_2 * Daily\ Mean\ Indoor\ Temperature_{ij} + \beta_2 * Heat\ Action\ Plan + b1\left(Building(participant)\right) + \varepsilon_{ij} \quad (2)$$
